# Field Determination of Phosphate in Environmental Water by Using a Hand-Powered Paper Centrifuge for Preconcentration and Digital Image Colorimetric Sensing

**DOI:** 10.1155/2022/7359197

**Published:** 2022-01-17

**Authors:** Zhen Pan, Xiaozhan Nong, Yajing Xie, Yan Li, Hui Zeng, Ying Liang, Min Zhang

**Affiliations:** School of Life and Environmental Sciences, Guilin University of Electronic Technology, Guilin, Guangxi 541004, China

## Abstract

Phosphate concentration in natural water has been used as a water quality indicator, as it is one of the major nutrients for aquatic plants. However, the traditional phosphomolybdenum blue (PMB) method has limited sensitivity for visual or camera-based detection, leading to underestimation of the phosphate concentration. We present an ultralow-cost, rapid field preconcentration and digital image colorimetric sensing of low-concentration phosphate method for water analysis. A novel hand-powered paper centrifuge (paperfuge) is used for sample preparation and preconcentration. This paperfuge is made of two circular paper discs and a string. Six centrifuge tubes (CTs) originally used as glue dispensing tips with a sample capacity of ∼230 *μ*L, are loaded on the paperfuge. After sampling, phosphate in the water sample is reacted to form PMB. Then, the reacted sample is drawn into a CT using an autopipette before the CT bottom is sealed by glue. After Oasis® HLB sorbents are added through the top of the CT, the CT top is also sealed with glue. The HLB sorbents adsorb PMB and are accumulated in the CT tip through centrifugation. The CT tips are cut and analyzed with the ImageJ software. It was found that the blue color intensity of sorbents is in a linear relationship to the phosphate concentration, with a linear range of 0–5 *μ*M (*r*^2^ = 0.9921) and limit of detection of 0.19 *μ*M. In addition, this method has been applied to in-field water analysis. The results are in agreement with the standard PMB method.

## 1. Introduction

Centrifugation is a commonly used technique to separate particles from the solution phase or fluids of different densities. This technique is widely applied in multiple areas, including diagnostic test assays [1, 2], biochemistry [3], and chemical separation [4], as an important step of sample preparation. For environmental analysis, the suspended sediment is removed or collected via centrifugation. Centrifugation is also used to help in precipitate separation in gravimetric analysis for the determination of macro- or major compounds in water samples. For measuring trace or ultratrace analytes in natural water, including metal ions [5, 6] and phosphate [4, 7], coprecipitation followed by centrifuging has been applied.

Due to its high-power consumption and bulky size, a centrifuge is normally considered as a laboratory-based instrument. Recently, lab-made low-cost centrifuges coupled with paper-based analytical devices (*μ*PADs) [8] or microchips [1, 2] have been reported for portable analysis. In 2017, inspired by children's toy, whirligig, Bhamla et al. [9] reported an ultralow-cost centrifuge (“paperfuge”) made from paper and string. This paperfuge is hand-powered, ultralow-cost, and easy-to-build with speeds up to 125,000 rpm (equivalent centrifugal forces of 30,000 g). It has been applied for point-of-care diagnostic testing in resource-limited environments. Further modification to increase the paperfuge loading capacity for molecular biology using a 3D-printing module has been applied [3].

Phosphate concentration in natural water has been used as a water quality indicator, as it is one of the major nutrients for aquatic plants. As the typical limiting nutrient in aquatic environments, phosphate concentration in natural water is much lower than nitrogen nutrients. According to the Redfield ratio, the ideal atomic ratio of N : P for balanced growth of aquatic plants is approximately 16 : 1. Therefore, even small changes in phosphate concentration may impact the water quality and biological condition significantly.

The phosphomolybdenum blue (PMB) method is widely applied for the determination of phosphate in natural water due to its easy-to-use feature [10, 11]. However, the formed PMB product is light blue with maximum absorption at the near-infrared region, resulting in poor visual or camera-based detection sensitivity. The lowest-reported limit of detection (LOD) of *μ*PADs based on the PMB reaction is 1.6 *μ*M for phosphate [12], while paper-based screen-printed electrochemical sensor has a LOD of 4 *μ*M [13]. Both methods are not able to provide enough sensitivity to the analysis of most of the unpolluted water. Therefore, the determination of trace phosphate in water by instrument-free or low-cost field approaches is a challenge.

In this work, we developed a hand-powered paperfuge for in-field analysis of phosphate at the sub-*μ*M (sub-ppb) level. In order to enhance the sensitivity, preconcentration of phosphate was performed by two approaches, one of which employed a cationic surfactant, cetyltrimethylammonium bromide (CTAB), to react with PMB forming an ion-paired precipitate (PMB-CTAB) [14], and the other used Oasis® HLB sorbents for the solid phase extraction (SPE) of PMB (PMB-SPE) [15–17]. A newly developed hand-powered paper centrifugation was applied for phosphate preconcentration. A smartphone equipped with microlens was used for rapid quantification. The demonstrated example showed that enhanced sensitivity for low-concentration phosphate was obtained via an ultralow-cost and easy-to-use approach.

## 2. Experimental Setup

### 2.1. Chemicals and Reagents

Analytical or higher-grade reagents were purchased from Aladdin, China, unless stated otherwise. R1 of the PMB reaction consisted of 25.2 g/L ammonium molybdate tetrahydrate (Sinopharm, China), 0.6 g/L potassium antimony tartrate, and 26.7% (v/v) sulfuric acid. R2 was 100 g/L ascorbic acid. The CTAB solution was 5 g/L. SPE sorbents were obtained from a Waters Oasis® HLB cartridge (200 mg, 6 cc, particle size 30 *μ*m). The sorbents were mixed with 20 mL water to form a suspension. The suspension was shaken before usage to obtain a homogeneous mixture.

### 2.2. Paperfuge

**Figure 1:**
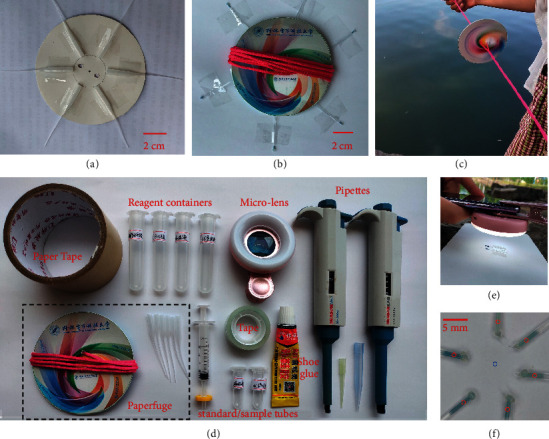
(a) Photo of taped six empty tubes on the paperfuge; (b) image of the paperfuge after preconcentrating phosphate; (c) spinning paperfuge; (d) materials required for field analysis by the PMB-SPE method; (e) capturing photo by a mobile phone equipped with a microlens; and (f) photo of six tube tips with preconcentrated PMB; cycles indicate the selected areas for analyzing color intensity.

The paperfuge was made by two circular paper discs (thickness 0.35 mm, radius, *r* = 50 mm) and a string (72 cm total length, folded in using) that passed through two holes around the center (1 cm away) of discs (Figure 1(a)). Centrifuge tubes were purchased from https://www.taobao.com, which was originally used as a gluing microtip with a maximum capacity of ∼230 *μ*L. The inner diameter of the tube tip is 0.5–0.6 mm, and the body total length is 66–71 mm. Six centrifuge tubes were attached around the paperfuge, sandwiched between the two paper discs (Figure 1(b)). The tube tip was 80 ± 5 mm away from the center. Two large plastic tubes (3 cm outer diameter) were used as handles held by a user during spinning (Figure 1(c)). The total rotation number in 30 s was measured via a digital tachometer (DT6236 C, Hongmei, China).

### 2.3. Sample Preparation and Phosphate Measurement

**Figure 2:**
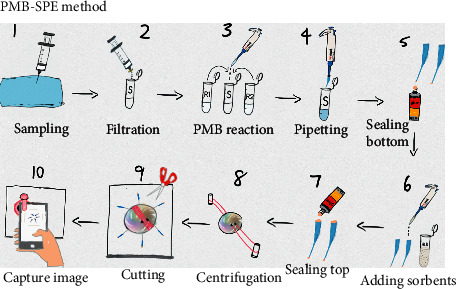
Schematic illustration of field preconcentration and digital image colorimetric sensing of phosphate. The description is detailed in Section 2.3.

Figure 2 demonstrates the procedure of the PMB-SPE method. The water sample was collected (step 1) and then filtrated by a 0.45 *μ*m membrane filter (step 2). R1 (0.4 mL) and R2 (0.3 mL) were added into 5 mL sample for PMB reaction (step 3). 120 *μ*L reacted solution was drawn into the centrifuge tube by using a 20–200 *μ*L autopipette (step 4); then, the tube tip was sealed by a waterproof glue (801, Weihao, China) in-field or hot glue in-lab (step 5). HLB suspension (30 *μ*L) was added into the tube (step 6). The top of the tubes was sealed with glue (step 7). The centrifuge tubes were taped and sandwiched between two paper discs. Step 8 shows the spinning of the paperfuge, which lasted for 30 s. In step 9, the tips with HLB sorbents were cut and placed around in a circle on a white paper for photographing (Figure 1(f)). A microlens kit (focusing range, 2.5–6 cm. NEPPT, China) equipped with white LEDs was incorporated with a smartphone (HUAWEI, China) for taking close-up photos in step 10 (Figure 1(e)). The procedures of the PMB-CTAB method are illustrated in Figure S1.

### 2.4. Image Analysis

The digital images were analyzed by ImageJ (http://imagej.nih.gov/ij/). An oval-shaped area was obtained with the mean of grey values (*GV*_tip_) at the middle of the tube tip (red cycles in Figure 1(f)). On the background paper, one of the other cycles (blue cycle in Figure 1(f)) was also analyzed to get a reference grey value (*GV*_ref_). The output (Δ*GV*) indicating the color intensity was calculated as follows:

(1) $$\begin{array}{c} \Delta GV=GV_{\text{ref}}-GV_{\text{tip}}. \end{array}$$

## 3. Results and Discussions

### 3.1. Configuration of the Paperfuge

The paperfuge consisted of two paper discs, a string, and 6 centrifuge tubes (Figure 1). Firstly, we compared different tubes and pipette tips to select the suitable centrifuge tube (Figure S2). Standard 1.5 mL microcentrifuge tubes were too heavy to achieve a high rotational speed by hand power. In addition, the precipitate would resuspend if the 1.5 mL microcentrifuge tube was turned upside down. Using a pipette tip of 200 *μ*L or less enabled us to collect PMB-CTAB precipitate in the tip by hand power but resuspension was still presented. Therefore, further narrowing the tip of the centrifuge tube was necessary. Microfabrication of a special design tube enabled us to address the issue. However, in order to reduce the cost and maintain accessibility, a low-cost off-shelf product, originally used for dispensing super glue, was selected.

**Figure 3:**
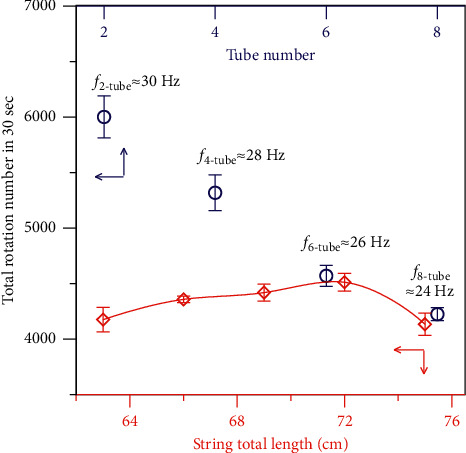
Effects of tube number (○, *n* = 6) and string length (◇, *n* = 3) to the total rotational number. Frequency (*f*), pulling on frequency.

In order to obtain a higher centrifugal force, the paper disc size, number of centrifuge tubes, and string length have been studied and optimized. The size of the paper disc was selected to be *r* = 50 mm, since a smaller size was inconvenient for taping tubes, while a larger size reduced the rotational speed under the same force [9]. The total rotational number in 30 sec of the attached centrifuge tube numbers of 2, 4, 6, and 8 were measured and compared (Figure 3). Less tube number allowed a higher rotational speed and hence a greater centrifugal force, but the sample throughput would be decreased. The pulling on frequency was decreased with more tubes as more weight was loaded, and six tubes gave a frequency (*f*) of ∼26 Hz. A tube of six with a total rotation of 4570 ± 95 rpm was finally selected as a compromise. Furthermore, the string length was compared at 63–75 cm, with the maximum speed obtained at 72 cm (Figure 3).

### 3.2. Effective Photographing Time

**Figure 4:**
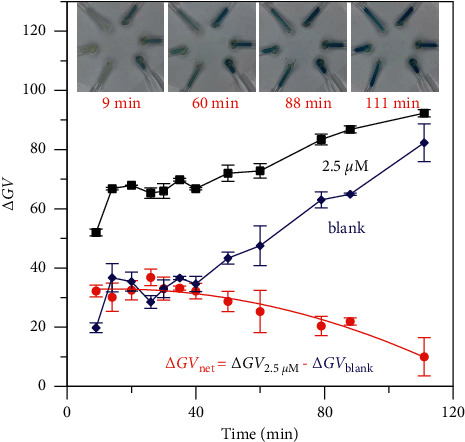
Effect of photographing time on the color intensities of 2.5 *μ*M standard and blank (PMB-HLB method, *n* = 3).

We found that PMB and PMB-CTAB ion-paired compounds turned darker gradually after reaction, which would affect the color intensity measurement. Therefore, the effective photographing time has been investigated. Three phosphate standards (2.5 *μ*M) and three blank samples were measured by the PMB-SPE method, photos were taken at intervals, and the color intensity as a function of time was obtained. As shown in Figure 4, the blue color of both standards and blanks were stable from 15 to 40 min but became significantly darker after 40 min. The difference of Δ*GV* (red cycle in Figure 4) between 2.5 *μ*M standard and the blank was decreased with enhanced color intensity, resulting in a loss of sensitivity and a decreased signal-to-noise ratio. The change of color should result from the reduction of excess molybdate under a condition of pH > 0.9 [18]. After the preconcentration of HLB sorbents, the tip was lack of solution, leading to changing of pH. Hence, we concluded that the digital image colorimetric detection had to be taken within the 15–40 min time frame after the addition of HLB.

### 3.3. Preconcentration of PMB-CTAB Ion Pair

We found that the centrifugation time for preconcentration of PMB-CTAB ion pair compound was longer than that of HLB sorbents. The centrifugation time of 3 min was required to complete the ion pair reaction for 150 *μ*L samples, while only 30 s was needed for HLB sorbents. In addition, the PMB-CTAB method includes a 10 min 35°C water bath step, which makes the entire analysis time of the PMB-CTAB method 13 min longer than that of the PMB-SPE method. As mentioned in section 3.2, the blue color was darkening along with time, which is thought to be due to the reduction of molybdate reagent. Our result also found that the darkening process was enhanced through the PMB-CTAB ion pair reaction, and a significant change of color was found right after the centrifugation. Therefore, the PMB-CTAB method gave a much higher sensitivity (3 to 5 times than the PMB-SPE method) with higher background (1.2 to 3 times higher). The slopes of three calibration curves obtained by the PMB-CTAB method within a day was 27.8 ± 12.0 (*r*^2^ = 0.949–0.965), while under the same conditions, the slopes of three calibration curves obtained by the PMB-SPE method was 7.92 ± 0.82 (*r*^2^ = 0.950–0.992). The larger uncertainty of the PMB-CTAB method, in longer analysis time and the requirement of water bath step, all together made this method less favorable for field analysis than the PMB-SPE method. Therefore, only the PMB-SPE method was selected for field application. It is worth mentioning that the cost of the CTAB reagent is much lower than the cost of the HLB sorbents. The method still could be useful for on-field preconcentration of phosphate, followed by analyzing the preconcentrated PMB compound in a laboratory.

### 3.4. Optimization of the PMB-SPE Method

**Figure 5:**
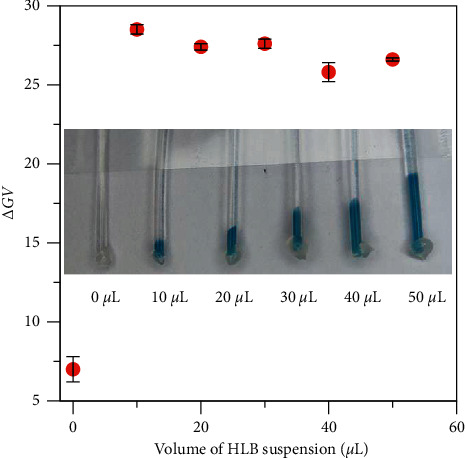
Effect of HLB suspension volume on the color intensity of 2.5 *μ*M standard (*n* = 3).

To further optimize the PMB-SPE method, the effects of reagents amount were investigated. In this section, the preparation of R1 and R2 was referred to the study by Pai et al. [19] and Drummond and Mather [18]. A ratio of [H+]/[MoO_4_^2-^] was fixed at 75 as recommended [18, 19]. 0.05 to 0.5 mL R1 was added into 5 mL sample, in which no significant change of signal output was found within this range. Therefore, R1 of 0.4 mL was selected, which is also recommended by other works [18, 19]. For R2, 0.1 to 0.5 mL was added, and the signal output was evaluated. It was found that the signal output of 2.5 *μ*M phosphate was proportional to the amount of R2 added. However, with more R2, the yellow color of the ascorbic acid in the solution became stronger, which affected the colorimetric detection. Therefore, 0.3 mL R2 was selected as a compromise. The volume of HLB suspension added into a centrifuge tube was studied from 0 to 50 *μ*L. As shown in the photo in Figure 5, more added HLB suspension generated a larger amount of blue precipitation in the CT tips but with negligible impact to the color intensity (red point). Finally, 30 *μ*L was selected, allowing enough space to pick up the color intensity of the precipitate and less HLB sorbent consumption. The added HLB sorbent was estimated to be 0.3 mg.

### 3.5. Analytical Performance of the PMB-SPE Method

**Figure 6:**
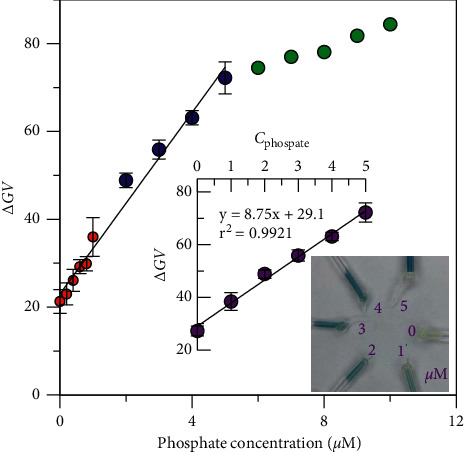
Calibration curves of the PMB-SPE method. Batches were color coded: red, 0–1 *μ*M (*n* = 3); blue, 2–5 *μ*M (*n* = 3); green, 6–12 *μ*M (*n* = 1); and purple (with photos), 0–5 *μ*M (*n* = 3).

The performance of the proposed PMB-SPE method was studied, including the linearity and reproducibility. Color intensity (as Δ*GV*) of phosphate standards at a range of 0–11 *μ*M was obtained by seven batches of centrifugation. Good linearity was obtained between 0 and 5 *μ*M (Figure 6), with *r*^2^ = 0.9921, though the linearity deteriorates over 5 *μ*M. Twelve blank samples from two batches of centrifugation were measured, providing a Δ*GV* value of 26.25 ± 0.54. The limit of detection (LOD, 3*σ*) was calculated as 0.19 *μ*M. The method reproducibility was obtained by measuring 18 standards of 2.5 *μ*M phosphate from three batches (*n* = 6 in each batch). The obtained Δ*GV* values in each batch were 48.8 ± 1.6, 48.2 ± 2.6, and 47.2 ± 2.5, with the relative standard deviations (RSDs) 3.24%, 5.97%, and 5.29%, respectively. The RSD between batches was 1.63%.

### 3.6. Interference Study

A study was conducted to investigate the influence of the possible interfering species in the water sample on the PMB-SPE method. The signal reading of 2.5 *μ*M phosphate was used as the reference. A potential interfering species (100 *μ*M) was added into the 2.5 *μ*M phosphate and measured via the same experimental procedure. The results of the interference study are given in Table 1. A typical anionic surfactant, sodium dodecyl sulfate (SDS) of 100 *μ*M, showed no interfere to the measurement, but CTAB of 100 *μ*M reduced the signal intensity to (84.9 ± 4.6)%.

Coexisting silicate and arsenate in the water sample may interfere with the determination of phosphate by using the PMB method [20]. In the reference studies [15, 17], the authors use the HLB cartridge to solid-phase extraction of PMB compound, and the influences of silicate and arsenate have been investigated to the method. Up to 200 *μ*M, silicate showed no significant interference [15, 17]. In this work, 100 *μ*M silicate was added into 2.5 *μ*M phosphate standard, and no interference was found, which agreed with the references. It has been reported that more than 100 nM arsenate could enhance the absorbance signal of the PMB method [15, 17]. As arsenate concentrations in unpolluted water are low, the influence could be ignored. In case the arsenate concentration is high, the addition of reducing reagents (e.g. L-cysteine) to convert arsenate to arsenite would eliminate the inference [15].

### 3.7. Application

We applied the PMB-SPE method in-field to water samples. The standard PMB method [21] was used as a reference method. The results are given in Table 2. The calculated *t* values were lower than the critical *t* value of 2.92 (*P*=0.10, *f* = 2), which indicates that the proposed method matched very well with the standard PMB method.

## 4. Conclusions

In summary, we proposed an easy-to-repeat field analysis method for phosphate. The method used off-the-shelf ultralow-cost components to obtain high sensitivity. The LOD of the PMB-SPE method is 0.19 *μ*M that is comparable or superior to automatic flow-analysis techniques [11]. The microcentrifuge tube containing preconcentrated phosphate can be mailed back to a laboratory and redissolved to verify the field analysis results [17].

## Figures and Tables

**Table 1:** Effects of possible interfering species (100 *μ*M) on measuring phosphate (2.5 *μ*M) via the PMB-SPE method (*n* = 3).

Compound	Relative sensitivity to phosphate (%)
Cetyltrimethylammonium bromide (CTAB)	84.9 ± 4.6
Sodium dodecyl sulfate (SDS)	98.5 ± 1.5
Ethylenediaminetetraacetic acid (EDTA)	79.1 ± 7.3
Silicate	98.4 ± 2.0

**Table 2:** Determination of water samples with the proposed PMB-SPE method and a standard PMB method.

Sample	PMB-SPE method, *μ*M^a^	Standard PMB method, *μ*M	*t* value^b^
Lake 1	ND	ND	—
Lake 2	2.50 ± 0.25	2.43	0.48
Lake 3	ND	0.06	-
Huajiang river	1.27 ± 0.12	1.15	1.73
Groundwater	0.30 ± 0.04	0.31	0.43

^a^Mean ± standard deviation, *n* = 3. ^b^*t* critical value (*P*=0.10, *f* = 2) = 2.92.

## Data Availability

The data used to support the finding of this study are available from the corresponding authors upon request.

## Supplementary Materials

Supplementary Materials Additional figures. Figure S1: schematic illustration of field preconcentration and digital image colorimetric sensing of phosphate by the PMB-CTAB method. Figure S2: centrifuge tubes used in this study. .
