# Supplementary material for: Field Determination of Phosphate in Environmental Water by Using a Hand-Powered Paper Centrifuge for Preconcentration and Digital Image Colorimetric Sensing
Source: J Anal Methods Chem. 2022 Jan 17;2022:7359197. doi: 10.1155/2022/7359197 (PMC8786554; doi:10.1155/2022/7359197)
Supplement: Supplementary Materials — Additional figures. Figure S1: schematic illustration of field preconcentration and digital image colorimetric sensing of phosphate by the PMB-CTAB method. Figure S2: centrifuge tubes used in this study. . [file 7359197.f1.pdf]

Supplementary Information

Field determination of phosphate in environmental water by using a hand-powered paper centrifuge for preconcentration and digital image colorimetric sensing

Zhen Pan#, Xiaozhan Nong#, Yajing Xie, Yan Li, Hui Zeng*, Ying Liang, Min Zhang*

School of Life and Environmental Sciences, Guilin University of Electronic Technology, Guilin, Guangxi, 541004

* Corresponding authors.

# These individuals (Z. Pan and X. Nong) had equal contributions to this work.

Email address:

zhangmin@guet.edu.cn (M. Zhang)

guetzenghui@foxmail.com (H. Zeng)

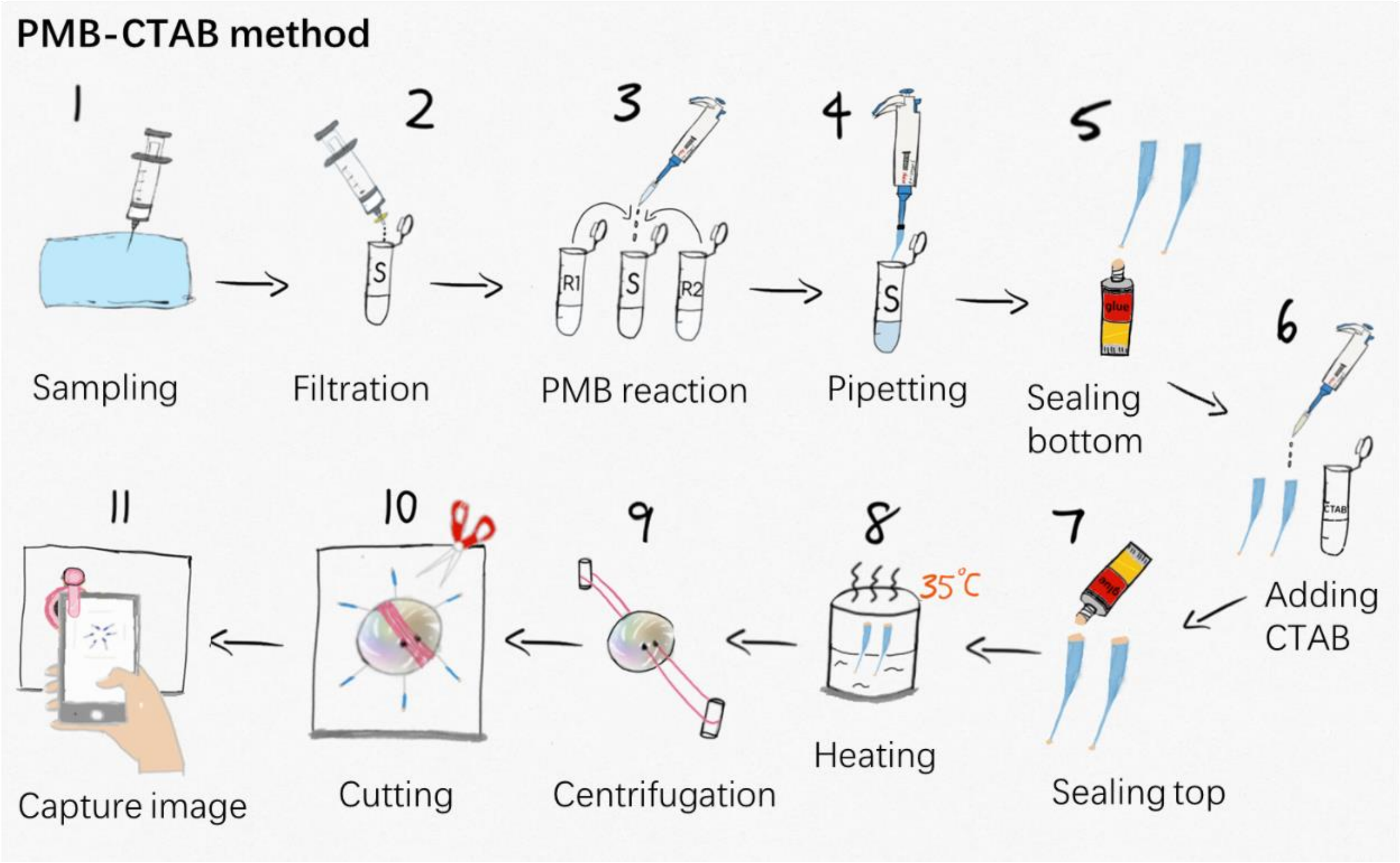

Figure S1. Schematic illustration of field preconcentration and digital image colorimetric sensing of phosphate by the PMB-CTAB method.

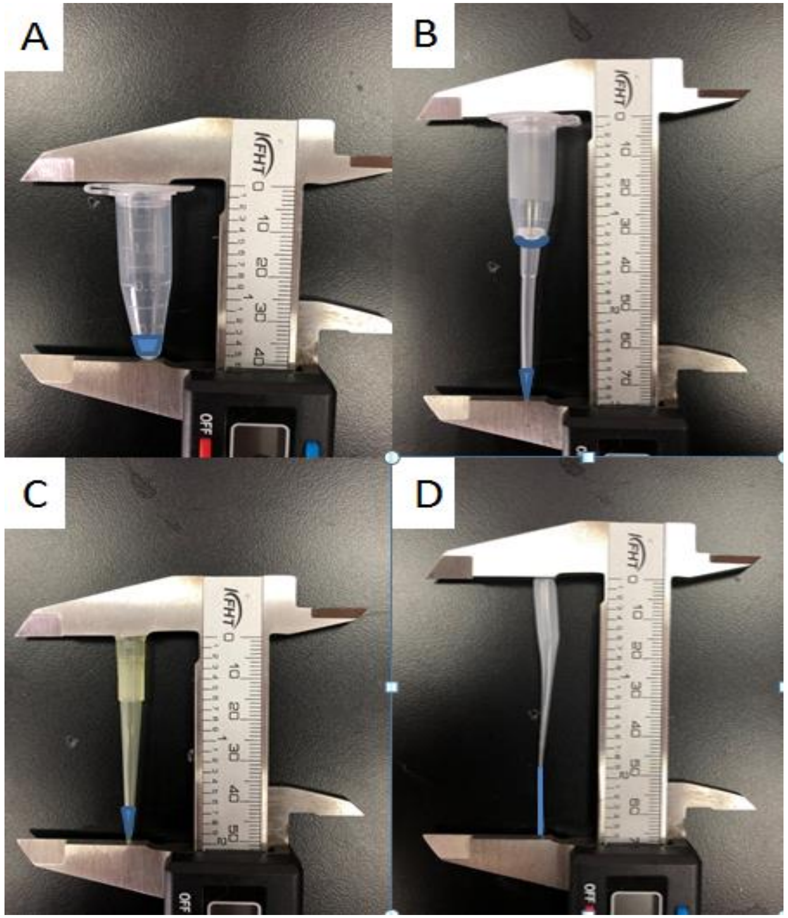

Figure S2. Centrifuge tubes used in this study. Blue blocks indicate the position of precipitate.
